# Absence of human T-cell lymphotropic virus type I and human foamy virus in thymoma

**DOI:** 10.1038/sj.bjc.6601841

**Published:** 2004-05-04

**Authors:** H Li, P J Loehrer, M Hisada, J Henley, D Whitby, E A Engels

**Affiliations:** 1 Viral Epidemiology Branch, Division of Cancer Epidemiology and Genetics, National Cancer Institute, Department of Health and Human Services, 6120 Executive Blvd., EPS 8010, Rockville, MD 20892, USA; 2 Indiana University School of Medicine and Indiana University Cancer Center, Indianapolis, IN, USA; 3 Viral Epidemiology Section, AIDS Vaccine Program, SAIC-Frederick, National Cancer Institute-Frederick, Frederick, MD, USA

**Keywords:** thymoma, human T-cell lymphotropic virus type I (HTLV-I), human foamy virus, simian foamy virus, retrovirus

## Abstract

The cause of thymoma, a rare malignancy of thymic epithelial cells, is unknown. Recent studies have reported the detection of DNA from human T-cell lymphotropic virus type I (HTLV-I) and human foamy virus (HFV) in small numbers of thymoma tumours, suggesting an aetiologic role for these retroviruses. In the present study, we evaluated 21 US thymoma patients and 20 patients with other cancers for evidence of infection with these viruses. We used the polymerase chain reaction to attempt to amplify viral DNA from tumour tissues, using primers from the *pol* and *tax* (HTLV-I) and *gag* and *bel1* (HFV) regions. In these experiments, we did not detect HTLV-I or HFV DNA sequences in any thymoma or control tissues, despite adequate sensitivity of our assays (one HTLV-I copy per 25 000 cells, one HFV copy per 7500 cells). Additionally, none of 14 thymoma patients evaluated serologically for HTLV I/II infection was positive by enzyme-linked immunoassay (ELISA), while five (36%) had indeterminate Western blot reactivity. In comparison, one of 20 US blood donors was HTLV-I/II ELISA positive, and nine (45%) donors, including the ELISA-positive donor, had indeterminate Western blot reactivity. Western blot patterns varied across individuals and consisted mostly of weak reactivity. In conclusion, we did not find evidence for the presence of HTLV-I or HFV in US thymoma patients.

Thymoma is a rare malignancy of thymic epithelial cells of unknown aetiology. Thymoma usually arises in middle-aged or elderly adults (Engels and Pfeiffer, 2003). The tumour is closely associated with several autoimmune conditions, especially myasthenia gravis (Thomas *et al*, 1999). Patients with thymoma are at increased risk of developing non-Hodgkin's lymphoma and perhaps other malignancies (Masaoka *et al*, 1994; Welsh *et al*, 2000; Engels and Pfeiffer, 2003).

Of interest, recent reports have suggested that two retroviruses could play a causal role in thymoma. DNA sequences from human T-cell lymphotropic virus type I (HTLV-I), the viral cause of adult T-cell leukaemia/lymphoma, were detected in tumour tissue obtained from 11 Italian thymoma patients (Manca *et al*, 2002). Notably, HTLV-I infection is rare in Italy, and none of the patients had obvious risk factors for infection. The other retrovirus possibly implicated in thymoma is human foamy virus (HFV), originally isolated from an African patient with nasopharyngeal carcinoma. Despite its name, HFV is not clearly a natural infection of humans (Meiering and Linial, 2001). Nonetheless, Saib *et al* (1994) identified HFV DNA sequences in peripheral blood mononuclear cells from a myasthenia gravis patient from the Indian Ocean island of Comoros. Subsequently, Liu *et al* (1996) amplified HFV DNA sequences from thymus tissue obtained from four Taiwanese patients with myasthenia gravis, two of whom had thymoma.

In the present US-based investigation, we sought to confirm the previously reported detection of these two retroviruses, HTLV-I and HFV, in thymoma tumours. We also evaluated patient sera for HTLV-I and HTLV-II antibodies.

## MATERIALS AND METHODS

### Patients

The study included archived tumour samples (stored at or below −70°C) from 21 thymoma patients treated at the Indiana University Cancer Center. Clinical data were unavailable for one patient. For the remainder, 10 were female, and the median age at thymoma diagnosis was 48 years (range 22–76). A total of 18 (90%) were white, two were black (10%), and all were born in and resided in the US. By World Health Organization histologic classification (Dadmanesh *et al*, 2001), subjects had subtypes A (*n*=1), B1 (*n*=9), B2 (*n*=6), B3 (*n*=3), and C (*n*=1). By Masaoka stage (Masaoka *et al*, 1981), subjects had tumours of stage I (*n*=5), II (*n*=8), III (*n*=2), IVa (*n*=4), and IVb (*n*=1). Four patients (20%) had myasthenia gravis, while five patients (25%) had a history of or developed additional cancers during clinical follow-up (one case each of prostate cancer, non-small-cell lung cancer, thyroid cancer, ovarian cancer, and glioblastoma). Serum samples were available for 14 of these 21 patients.

For control tissues, we evaluated archived tumour samples (stored at −70°C) from US patients with colon cancer (five cases), breast cancer (seven), or lung cancer (eight). For antibody studies, we used sera from 20 US blood donors as controls. All specimens were anonymised and tested only after delinking from clinical information.

### Molecular detection of HTLV-I and HFV

DNA extraction and polymerase chain reaction (PCR) experiments were performed simultaneously for thymoma cases and controls. DNA was extracted from tissues using the DNeasy Tissue Kit (QIAGEN, Valencia, CA, USA) according to the manufacturer's instructions. Briefly, for each specimen, 15–20 mg of minced tissue was lysed overnight in Tissue Lysis Buffer and 20 *μ*l proteinase K solution (20 mg ml^−1^) at 55°C. Subsequently, 4 *μ*l of RNase A solution (100 mg ml^−1^) was added and incubated at 37°C for 10 min. The mixture was then placed on a QIAamp spin column. DNA was eluted after two wash steps, and spectrophotometry was used to quantify DNA and assess its purity. DNA yield was similar for thymoma tissues and control specimens (median 34 *μ*g, range 3–212 from thymoma specimens, *vs* median 27 *μ*g, range 12–61 from control specimens; *P*=0.51). Extracted DNA was of high purity (median OD_260_/OD_280_ ratio 1.86, range 1.72–1.99 for thymoma specimens; median 1.83, range 1.76–1.89 for control specimens).

For PCR detection of HTLV-I and HFV, we used primers from the *pol* and *tax* (HTLV-I) and *gag* and *bel1* (HFV) regions. Specifically, HTLV-I sequences were amplified using SG231/SG238 for *pol* (239 bp product, nucleotide position 2802–3038) (Ehrlich *et al*, 1990) and SK43/SK44 for *tax* (161 bp product, nucleotide position 7359–7517) (Saito and Ichijo, 1992; Manca *et al*, 2002). Human foamy virus sequences were amplified using NC#8/PR#2 for *gag* (504 bp product, nucleotide position 3354–3855) (Yu *et al*, 1999) and #5′bel1/#3′bel1 for *bel1* (704 bp product, nucleotide position 10 182–10 883) (Yu *et al*, 1996). In each PCR experiment, 500 ng DNA was used as template in a 50 *μ*l reaction volume containing 1 × PCR buffer (Perkin-Elmer, Branchburg, NJ, USA), 1.5 mM MgCl_2_, 250 *μ*M of each dNTP (Gibco/BRL, Grand Island, NY, USA), 2.5 U of AmpliTaq Gold polymerase (Roche, Indianapolis, IN, USA), and 25 pmol of each primer. Polymerase chain reaction amplification was performed as follows: preheat at 95°C for 10 min, 40 cycles of amplification (95°C 30 s, 58°C 1 min, 72°C 30 s), followed by a final elongation step at 72°C for 10 min. Amplified products were analysed by 2% agarose gel electrophoresis with ethidium bromide staining. Positive controls were serial 10-fold dilutions of DNA from the HTLV-I-infected T-cell line MT1 or HFV plasmid pcHSRV2/2 (gift of Dr O Herchenröder, Dresden, Germany), calculated to contain 3 × 10^4^–3 × 10^−1^ copies of HTLV-I DNA or 10^6^–10^1^ copies of HFV DNA per reaction, respectively.

Protective clothing, dedicated equipment, newly prepared reagents and primers, and UV irradiation were used to prevent PCR contamination. Sample preparation, PCR amplification, and postamplification processing were performed in separate rooms. Additionally, to prevent contamination by laboratory controls, all PCR experiments were performed twice: the first PCR experiments were performed in the absence of positive controls and titration series (‘suicide’ PCR) (Raoult *et al*, 2000), while the second PCR experiments were subsequently repeated in the presence of positive controls to confirm the initial results. In a separate experiment (data not shown), we confirmed that samples contained adequate amounts of amplifiable human DNA using a quantitative PCR assay for human endogenous retrovirus 3 (Yuan *et al*, 2001).

### HTLV serology

Sera were tested for HTLV-I and HTLV-II antibodies by enzyme-linked immunoassay (ELISA; Dupont, Wilmington, DE, USA) and Western blot (HTLV Blot 2.4, Genelabs Technologies, Singapore) according to the manufacturer's protocol. Western blot bands were scored on a grey scale from 0 (absent) to 10 (maximum intensity). For HTLV-I, Western blot reactivity was defined as the presence of bands (2+ intensity) for recombinant gp46I and gp21 (*env* proteins) and p19 and p24 (*gag* proteins). For HTLV-II, bands of 2+ intensity for recombinant gp46II (*env*) and p24 (*gag*) were considered diagnostic. Weak reactivity (intensity = 1 on the gray scale) on these bands or reactivity only for other viral proteins was considered indeterminate for HTLV-I/II infection.

## RESULTS

**Figure 1:**
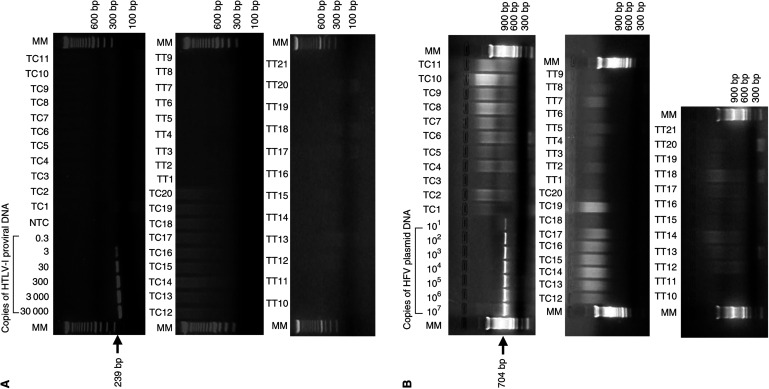
PCR amplification of HTLV-I and HFV sequences from thymoma and control tumour tissues. (**A**) Ethidium bromide stained gels of PCR products corresponding to HTLV-I *pol* region from a single experiment, obtained using the SG231/SG238 primer set. Gels include lanes for molecular size markers (MM), a titration series of HTLV-I DNA from MT1 cells (3 × 10^4^–3 × 10^−1^), no-template control (NTC), 20 tumour tissue controls (TC1-TC20), and 21 thymoma tissues (TT1-TT21). (**B**) Ethidium bromide stained gels of PCR products corresponding to HFV *bel1* region from a single experiment, obtained using #5′bel1/#3′bel1 primer set. Gels include lanes for MM, a titration series of HFV plasmid DNA (10^7^–10^1^ copies), 20 tissue controls (TC1-TC20), and 21 thymoma tissues (TT1-TT21). Arrows show the size of amplified products (239 bp for HTLV-I *pol*, 704 bp for HFV *bell*).

Using DNA extracted from thymomas, PCR amplification experiments for HTLV-I (*pol* and *tax*) and HFV (*gag* and *bel1*) were all negative. These experiments were performed twice for each specimen (once without positive controls, followed by once with positive controls), with complete concordance. Figure 1

shows results from experiments with positive controls, using primers from HTLV-I *pol* (panel A) and HFV *bel1* (panel B). In these experiments, serial dilutions of the positive control DNA demonstrated that PCR could identify three HTLV-I copies and 10 HFV copies per reaction (Figure 1).

**Figure 2:**
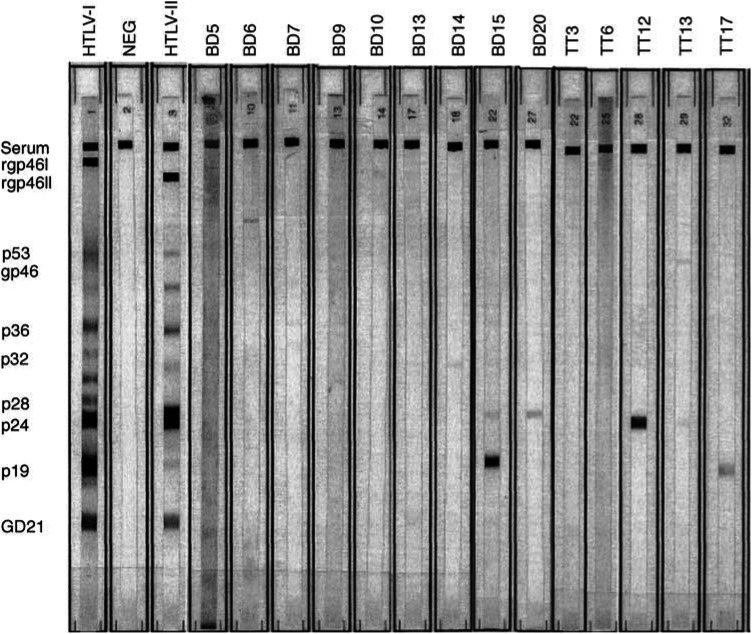
HTLV-I/II Western blot results. Results are shown for the nine blood donors (BD) and five thymoma patients (TT) with indeterminate Western blot results. Columns on the left correspond to positive controls for HTLV-I and HTLV-II and a negative control (NEG). Bands are indicated to the left of the figure and include a positive serum control (serum), recombinant gp46I and gp46II (rgp46I and rgp46II), and p21 (GD21). Bands were scored 0–10 on a grey scale (see Materials and Methods); bands scoring 1–2 on this scale are faint and may not be visible in this photograph. For instance, Western blot reactivity was noted for subject BD15 for bands rgp46I (grey scale level 1), rgp46II (1), p24 (5), p19 (9), and GD21 (1), while for subject TT13 only faint reactivity was noted for bands p53 (grey scale level 2) and p24 (1).

By ELISA, 14 of 14 thymoma patients and 19 of 20 blood donor controls were HTLV-I/II seronegative, while one blood donor (BD15) was HTLV-I/II seropositive. By Western blot, none of the 35 evaluated subjects was HTLV-I or HTLV-II seropositive. Indeterminate Western blots were observed in five thymoma patients (36%) and nine blood donors (45%); in most of these subjects, reactivity was weak (Figure 2

). Among thymoma patients with indeterminate Western blots, two had reactivity to *env* but not *gag*, and three had reactivity to *gag* but not *env*. Among blood donors, five had reactivity to *env* but not *gag*, and one had reactivity to *gag* but not *env*. Two blood donors (BD5 and BD15) had reactivity to both *gag* and *env* (but this reactivity did not meet our criteria for Western blot positivity), and one donor had reactivity only to Western blot proteins other than *gag* and *env*.

## DISCUSSION

HTLV-I and *foamy viruses* infrequently cause infections in humans (Schweitzer *et al*, 1995; Ali *et al*, 1996; Anonymous, 1996) but can integrate into and disrupt the host genome, making them potentially attractive agents for causing rare cancers such as thymoma. Nonetheless, using samples obtained from US patients, we were unable to confirm prior reports that either HTLV-I or HFV DNA is present in thymoma tumour tissue (Saib *et al*, 1994; Liu *et al*, 1996; Manca *et al*, 2002).

The single prior report of HTLV-I detection in thymoma was from Italy (Manca *et al*, 2002). In that study, Manca *et al* tested thymic tissue from 27 patients with myasthenia gravis (12 with thymoma, 15 with thymic hyperplasia). A DNA sequence corresponding to the HTLV-I regulatory gene *tax* was amplified from most cases (92% of thymomas, 93% of thymic hyperplasia specimens), whereas DNA corresponding to the structural gene *pol* was found in fewer tissues (75% of thymomas, 40% of thymic hyperplasia specimens). Additionally, sera from 83 other myasthenia gravis patients were studied by the same group (Manca *et al*, 2002). All were negative for HTLV-I/II antibodies by ELISA. A total of 17 sera were evaluated further by Western blot, and all showed antibody reactivity against recombinant HTLV-I/II p21 envelope protein, consistent with an indeterminate HTLV-I serology (only two showed reactivity against the *gag* protein p19).

The reasons why our findings regarding HTLV-I, which were convincingly negative, differ from those of Manca *et al* are unclear. Although a limitation of our study was its small size and the selection of patients from a single referral institution, our study included thymoma patients from various demographic categories and tumour subtypes. We amplified the same two HTLV-I gene regions as Manca *et al* did, and one primer set (SK43/SK44) was the same in both studies. In our experiments, we would have detected as few as three HTLV-I *pol* or *tax* copies in 500 ng genomic DNA, equivalent to one copy per 25 000 cells, had the virus been present. Thus, our assays were sufficiently sensitive to rule out the presence of HTLV-I in these specimens.

Similarly, we did not find evidence for a specific HTLV-I or HTLV-II antibody pattern in thymoma patients. One blood donor control had a positive ELISA result and indeterminate Western blot, suggesting that he might have been exposed to or infected with HTLV-I or HTLV-II. All other subjects were ELISA negative, and overall, equivalent proportions of thymoma patients and blood donor controls manifested indeterminate Western blots. In our study, p21 seroreactivity was weak and observed in only one thymoma patient (7%, in contrast to Manca *et al*) and four blood donors (20%). The limited-spectrum and mostly weak-intensity Western blot reactivity, coupled with the negative ELISA results, indicates that thymoma patients were infected with neither HTLV-I nor HTLV-II.

Furthermore, there is little epidemiological evidence to relate thymoma to HTLV-I infection. HTLV-I is endemic in some areas of Japan, and interestingly, thymoma is more frequently diagnosed in the US among Asians/Pacific Islanders than in other racial groups (Engels and Pfeiffer, 2003). However, the excess of thymoma in the US is not limited to Japanese (National Cancer Institute Surveillance, Epidemiology, and End Results data, authors' unpublished analyses), and the heightened thymoma incidence in Asians/Pacific Islanders might be due to other genetic or environmental factors. We considered the possibility that we missed HTLV-I in thymoma because HTLV-I infection is uncommon in the US (Anonymous, 1996). Nonetheless, HTLV-I is equally rare in Italy (Anonymous, 1996), and none of the cases studied by Manca *et al* (2002) had recognizable risk factors for acquiring HTLV-I.

Foamy viruses infect many mammal species, but no foamy virus uniquely infecting humans has been identified (Meiering and Linial, 2001). Based on extensive nucleotide and amino-acid homology (Herchenröder *et al*, 1994), the foamy virus originally isolated from a human (ie, ‘human foamy virus’) may actually be identical to a chimpanzee simian foamy virus isolate (SFVcpz). Indeed, the African patient from whom HFV was first isolated could have acquired the virus from a primate (Meiering and Linial, 2001), since such transmission of SFV can occur through close animal contact (Schweitzer *et al*, 1997; Heneine *et al*, 1998; Sandstrom *et al*, 2000; Brooks *et al*, 2002). Alternatively, the initial report may have represented a laboratory contamination (Meiering and Linial, 2001). Two large serosurveys subsequently failed to find HFV infection in diverse human populations (Schweitzer *et al*, 1995; Ali *et al*, 1996). In recognition of the likely identity of HFV with SFVcpz, some investigators refer to HFV as ‘SFVcpz(hu)’ (Meiering and Linial, 2001). Although evidence for infection with HFV (or a closely related primate virus) has been reported in patients with a range of autoimmune or idiopathic diseases, including Graves' disease, thyroiditis de Quervain, and multiple sclerosis, later studies cast doubts on those findings (Meiering and Linial, 2001). Documented human infection with SFV has not been linked to disease (Schweitzer *et al*, 1997; Heneine *et al*, 1998; Brooks *et al*, 2002).

There are sparse data regarding a possible role for HFV in thymoma or myasthenia gravis. Saib *et al* (1994) studied eight patients with myasthenia gravis, only one of whom (a female from Comoros) had HFV DNA sequences detected by PCR in peripheral blood mononuclear cells. On sequencing, part of the HFV *bel1* gene was deleted, suggesting the presence of a replication-incompetent variant of the virus. Additionally, serum from the patient reacted to multiple HFV antigens by Western blot and immunofluorescence assays. Liu *et al* (1996) reported amplifying HFV *gag* and *bel2* sequences from thymus tissue of four Taiwanese patients with myasthenia gravis (two with ‘lymphoepithelioma’ variants of thymoma/thymic carcinoma, two with thymic hyperplasia). All four cases also had low-titer neutralising antibody against HFV. Attempts in both studies to isolate HFV were unsuccessful (Saib *et al*, 1994; Liu *et al*, 1996). Neither study provided an explanation for how these individuals might have become infected with a rare human or primate virus.

Using primers designed to detect HFV sequences, we could not confirm these prior reports for US thymoma patients. With the same *bel1* primer set used by Saib *et al* (1994), we ruled out the presence of HFV DNA at a level of one copy per 7500 cells in tumour tissue obtained from US patients with thymoma. With similar sensitivity, we excluded the presence of HFV *gag* sequences. Our *gag* primers should also have been able to amplify SFVcpz DNA, since the 3′ primer (PR#2) perfectly matches the published SFVcpz *gag* sequence (Herchenröder *et al*, 1994), while the 5′ primer (NC#8) matches SFVcpz over its 3′ end for 16 contiguous nucleotides.

In conclusion, we did not find evidence for HTLV-I or HFV infection in US thymoma patients. It would be of further interest to study the relationship between HTLV-I, thymoma, and myasthenia gravis in geographic areas where HTLV-I is endemic. Since the cause of thymoma is unknown, further searches for a viral aetiology may be warranted.
